# Trust in Artificial Intelligence–Based Clinical Decision Support Systems Among Health Care Workers: Systematic Review

**DOI:** 10.2196/69678

**Published:** 2025-07-29

**Authors:** Hein Minn Tun, Hanif Abdul Rahman, Lin Naing, Owais Ahmed Malik

**Affiliations:** 1 PAPRSB Institute of Health Sciences, Universiti Brunei Darussalam, Core Residential, Tower 4, Room 201A, UBDCorp, Jalan Tungku Link, Bandar Seri Begawan, BE1410, Brunei Darussalam, 673 7428942; 2 School of Digital Science, Universiti Brunei Darussalam, Bandar Seri Begawan, Brunei Darussalam

**Keywords:** trust in artificial intelligence, decision support systems, health care workers, PRISMA

## Abstract

**Background:**

Artificial intelligence–based clinical decision support systems (AI-CDSSs) have enhanced personalized medicine and improved the efficiency of health care workers. Despite these opportunities, trust in these tools remains a critical factor for their successful integration into practice. Existing research lacks synthesized insights and actionable recommendations to guide the development of AI-CDSSs that foster trust among health care workers.

**Objective:**

This systematic review aims to identify and synthesize key factors that influence health care workers’ trust in AI-CDSSs and to provide actionable recommendations for enhancing their trust in these systems.

**Methods:**

We conducted a systematic review of published studies from January 2020 to November 2024, retrieved from PubMed, Scopus, and Google Scholar. Inclusion criteria focused on studies that examined health care workers’ perceptions, experiences, and trust in AI-CDSSs. Studies in non–English languages and those unrelated to health care settings were excluded. Two independent reviewers followed the Cochrane Collaboration Handbook and PRISMA (Preferred Reporting Items for Systematic Reviews and Meta-Analyses) 2020 guidelines. Analysis was conducted using a developed data charter. The Critical Appraisal Skills Programme tool was applied to assess the quality of the included studies and to evaluate the risk of bias, ensuring a rigorous and systematic review process.

**Results:**

A total of 27 studies met the inclusion criteria, involving diverse health care workers, predominantly in hospitalized settings. Qualitative methods were the most common (n=16, 59%), with sample sizes ranging from small focus groups to cohorts of over 1000 participants. Eight key themes emerged as pivotal in improving health care workers’ trust in AI-CDSSs: (1) System Transparency, emphasizing the need for clear and interpretable AI; (2) Training and Familiarity, highlighting the importance of knowledge sharing and user education; (3) System Usability, focusing on effective integration into clinical workflows; (4) Clinical Reliability, addressing the consistency and accuracy of system performance; (5) Credibility and Validation, referring to how well the system performs across diverse clinical contexts; (6) Ethical Consideration, examining medicolegal liability, fairness, and adherence to ethical standards;(7) Human Centric Design, pioritizing patient centered approaches; (8) Customization and Control, highlighting the need to tailor tools to specific clinical needs while preserving health care providers’ decision-making autonomy. Barriers to trust included algorithmic opacity, insufficient training, and ethical challenges, while enabling factors for health care workers’ trust in AI-CDSS tools were transparency, usability, and clinical reliability.

**Conclusions:**

The findings highlight the need for explainable AI models, comprehensive training, stakeholder involvement, and human-centered design to foster health care workers’ trust in AI-CDSSs. Although the heterogeneity of study designs and lack of specific data limit further analysis, this review bridges existing gaps by identifying key themes that support trust in AI-CDSSs. It also recommends that future research include diverse demographics, cross-cultural perspectives, and contextual differences in trust across various health care professions.

## Introduction

The adoption of artificial intelligence (AI) in health care has a potentially transformative impact on health care workers by enabling advancements in diagnostics, treatment planning, and patient management, thereby improving the health care system. The increasing availability of digitalized health care data, along with technological advancements in machine learning and deep learning algorithms, has enhanced the potential of AI-based clinical decision support systems (CDSSs). These systems can assist can health care workers by predicting patient outcomes and recommending optimal interventions, contributing to personalized medicine and improved health care efficiency [1,2]. Despite these advancements, health care professionals’ trust in AI-based CDSS (AI-CDSS) tools remains a critical factor for their successful integration and effective use in clinical practice. Furthermore, hesitation persists, particularly among highly skilled professionals, regarding AI’s ability to provide substantial clinical value [2-4].

Trust is a complex construct that affects how health care workers interact with AI-driven systems, which are developed through the complex and opaque mathematical mechanisms of machine learning models [2]. Trust holds value only when directed toward agents or systems that are genuinely reliable, as placing trust in untrustworthy sources can lead to severe, even life-threatening, consequences. It can be understood through 3 interconnected elements: belief in the truthfulness of claims (such as trusting the accuracy of advice), confidence in commitments (like relying on a bank to send monthly statements), and faith in competence (for example, trusting a dentist to carry out a procedure properly) [5]. Without adequate trust, health care workers may disregard AI recommendations, undermining the potential benefits of AI in enhancing patient care and optimizing clinical workflow.

Clinicians’ concerns about the opacity of AI decision-making processes, the potential for algorithmic bias, and the fear of technology replacing human judgment can undermine trust in these systems [6-9]. Furthermore, trust in AI-based systems is not a static concept; it evolves as health care workers interact with the technology and gain experience with its functionality and outcomes. Vereschak et al [10] highlight the importance of integrating theoretical elements of trust, such as vulnerability, positive expectations, and attitude, into the understanding of human-AI trust. Trust can also be reflected in behavioral dimensions, including decision time, reliance on or accepting recommendations, and compliance behaviors such as requesting recommendations. These behaviors have been conceptualized as passive indicators of trust, such as immediate agreement, disagreement, or mild agreement with the system’s recommendations, and can offer valuable insights into the level of trust and how it influences decision-making [11].

A growing body of research has explored trust in AI-CDSS tools from various perspectives, including those of clinicians, nurses, and pharmacists. These studies have examined trust through multiple lenses, ranging from algorithmic development and mathematical considerations, to the use of devil’s advocate approaches with large language models such as ChatGPT, to qualitative explorations of health care workers’ perspectives through a sociotechnical lens. Other angles include AI confidence levels and the impact of technology-induced dehumanization in health care. Trust has also been studied in the context of upstream relationships among different stakeholders [4,8-10,12]. Several factors influencing trust have been identified, including transparency, explainability, interpretability, privacy, ethical concerns, and the actionability or contestability required by decision makers. Additionally, the attitudes, perceptions, and individual experiences of health care workers have also been recognized as critical elements shaping trust [10-13].

Despite these findings, there appears to be a lack of synthesized insights and recommendations regarding the factors that influence health care workers’ trust in AI-CDSSs. Our study aims to fill this gap by systematically reviewing the literature to identify the constraints and facilitators of trust in AI-CDSSs. Guided by existing research, we intend to formulate practical recommendations for the design and implementation of AI systems that are trusted and accepted by health care practitioners. This research will contribute to the development of strategies that promote the use of AI-CDSSs in health care in a way that complements, rather than disrupts, clinical decision-making.

## Methods

### Review Design

Our systematic review follows the Cochrane Collaboration Handbook [14,15] and reports findings in accordance with the PRISMA (Preferred Reporting Items for Systematic Reviews and Meta-Analyses) 2020 checklist [16]. This review systematically consolidates findings from the past 5 years to address underexplored areas of health care workers’ trust in AI-CDSSs, identifying both enablers and barriers within trust dynamics [14-16]. The Critical Appraisal Skills Programme (CASP) tool [17] was used to assess the quality of included studies and the risk of bias.

### Literature Search Strategies

We conducted a systematic search of published studies focusing on trust in AI-CDSS between January 1, 2020, and November 30, 2024, guided by PRISMA guidelines with the PICO (population, intervention, comparison, and outcome) framework [14,16]. This study period was chosen to reflect the advancements and increased investment in AI, particularly following the release of generative models such as ChatGPT in the health care sector, especially in the aftermath of the COVID-19 pandemic. Our sources included PubMed, Scopus, and Google Scholar. The search strategy used a combination of English keywords, including “trust” or “acceptance” or “perception” and “artificial intelligence” or “AI” and “decision support systems” or “clinical decision support” or “AI-based decision support” and “healthcare workers” or “clinicians” or “nurses” or “medical professionals” or “healthcare providers.” Publication date filters were applied to include only studies within the specified time frame. Additionally, we used a snowball strategy to identify further sources from the references of relevant full texts. Medical Subject Headings (MeSH) and free-text terms were used to maximize search sensitivity and ensure comprehensive coverage of relevant literature, as described in Multimedia Appendix 1.

### Eligibility Criteria

We included research articles that explicitly described health care professionals’ trust, acceptance, or reliance on AI-CDSSs, specifically within clinical and primary care settings. Qualitative, quantitative, and mixed-method studies were all considered, provided they explored aspects of trust or acceptance among health care workers. We excluded studies unrelated to the relationship between trust in health care providers and AI-CDSSs. To maintain the scientific credibility of our review, we also excluded non–peer-reviewed articles, editorials, opinion pieces, and other forms of nonresearch literature.

### Data Extraction

Two researchers (HMT and HAR) initially screened titles and abstracts to determine whether they met the inclusion criteria. After removing duplicates, full texts were reviewed to assess potential exclusion criteria. Any disagreements regarding eligibility criteria were resolved through discussion with another team member. The Mixed Methods Appraisal Tool was used to assess the quality of studies, enabling evaluation across diverse methodological approaches [18]. We extracted key study details, including author, country of data origin, study design, and the type of AI method utilized. Additional information was systematically extracted for each study, focusing on the type of AI application, the role of health care workers, the study setting and location, the specific department or clinical focus, and the type of AI-based decision support system. We also recorded information on trust measurement tools, qualitative questions related to trust, assessed trust factors, trust-related outcomes, levels of trust observed, influencing factors, study limitations, conclusions or recommendations, and funding sources. Additionally, qualitative information, such as quotes, themes, and findings from interviews, focus groups, and open-ended survey responses, was extracted. The quality of the included studies was also evaluated based on the alignment between study objectives and results.

### Data Synthesis and Analysis

Data synthesis and analysis were conducted to systematically integrate and interpret the findings. Relevant data were organized into an evidence matrix using a standardized template in Google Sheets (Google LLC/Alphabet Inc). Advanced tools for systematic review and data extraction, including Zotero 6 (Corporation for Digital Scholarship), Elicit (Ought), and Rayyan (Qatar Computing Research Institute), were used to screen and analyze abstracts from 27 studies that met the inclusion and exclusion criteria. A comprehensive data charter was developed to summarize the characteristics of the included study [19]. This data charter includes the year of publication, geographic location, study setting, characteristics of CDSSs, methods used to evaluate trust, descriptions of the AI-CDSSs, and evaluation of trust-related factors in AI-CDSSs. Furthermore, we extracted qualitative outcomes and corresponding codes related to trust in AI-CDSSs. The extracted data were organized and analyzed in Microsoft Excel, with recurrent patterns categorized into themes using mind-mapping techniques. Each theme was carefully interpreted and synthesized into enablers and barriers to trust in AI-CDSSs. Based on these themes, we developed actionable recommendations for practical implementation, policy, and further research by triangulating insights from thematic synthesis, extracted quotes, and relevant literature.

### Quality and Risk of Bias Assessment

The quality of qualitative studies and the qualitative components of mixed-methods studies were assessed using the CASP tool. Each question in the tool was evaluated with 1 of 3 responses: “yes,” “no,” or “cannot determine.” Rather than producing a summative score, the CASP tool provides an overall assessment, categorizing studies as “not valuable,” “semivaluable,” “valuable,” or “very valuable,” as documented in previous literature. These assessments were based on a judgmental approach, in which reviewers evaluated the relevance and contribution of each study to understanding interaction traits within the AI-clinician quality of interaction construct [17]. Quality assessments were conducted independently by 2 reviewers (OAM and LN), with any disagreements resolved through consensus. This process ensured a rigorous and transparent evaluation of study quality.

## Results

### Study Selection

**Figure 1.**
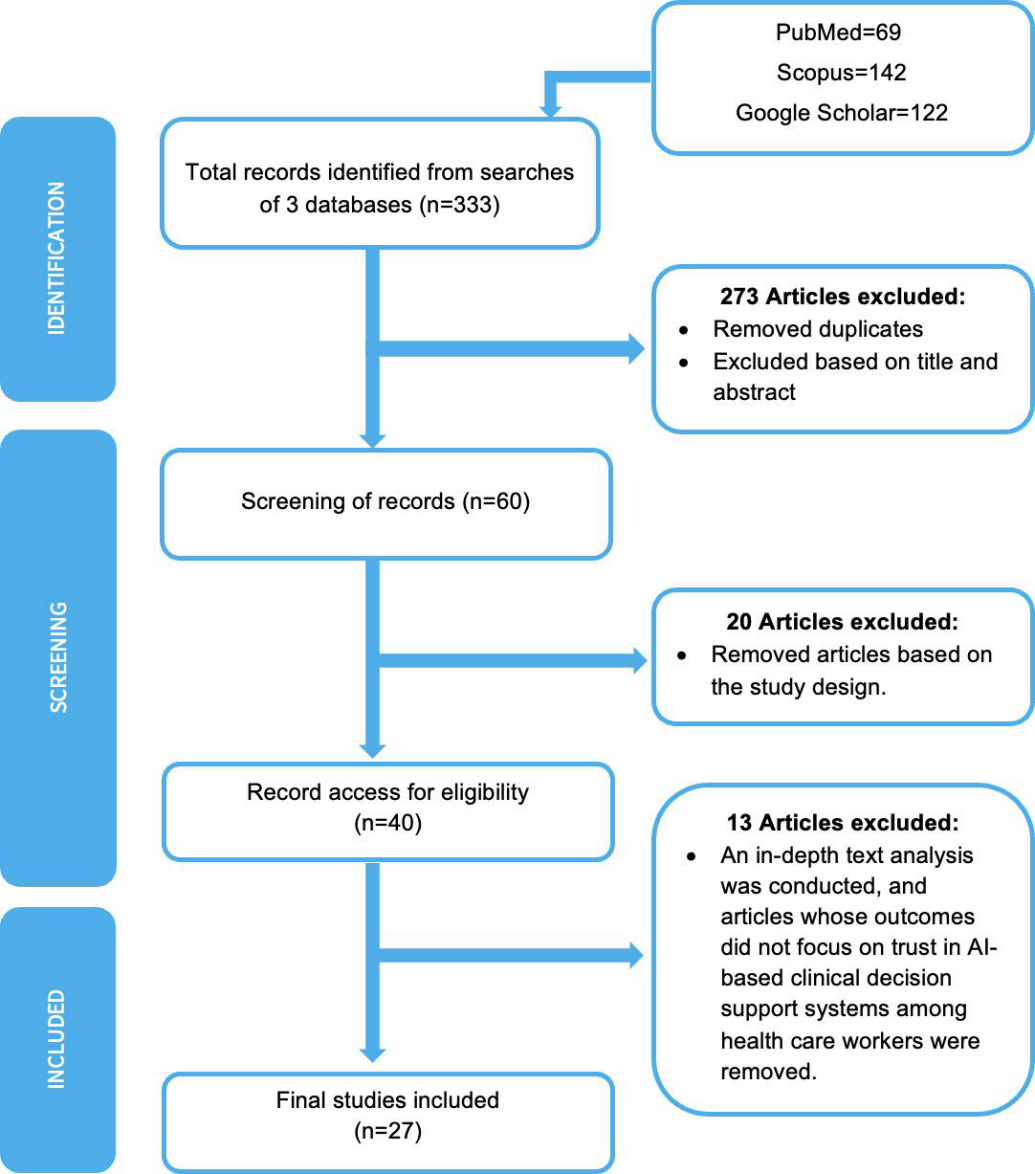
PRISMA (Preferred Reporting Items for Systematic Reviews and Meta-Analyses) flowchart for the study selection process. AI: artificial intelligence.

The article selection process consisted of 2 phases: (1) a review of titles and abstracts and (2) a full-text review. Figure 1 illustrates the study selection process. Initially, 333 records were identified from 3 databases: PubMed (69 records), Scopus (142 records), and Google Scholar (122 records). An independent reviewer screened these records to remove duplicates and articles deemed irrelevant based on titles and abstracts, resulting in 60 records advancing to the next stage. Further screening excluded 20 articles due to unsuitable study designs, leaving 40 for eligibility assessment. Following an in-depth full-text review and final discussions among the reviewers, 13 articles were excluded for lacking a focus on trust in AI-based decision support systems among health care workers. Ultimately, 27 studies were included in the final analysis.

### Quality Assessment of Included Studies

A total of 23 studies were assessed using the CASP checklist for qualitative analysis (Table 1), while 4 studies used other methodologies. Among the 23 studies, the majority (n=19, 83%) received a “Yes” rating for most CASP criteria and were categorized as “valuable” or “very valuable” in the quality assessment. Studies categorized as “semivaluable” (n=4, 17%) were flagged for issues such as the inappropriate use of qualitative methods to measure nonsubjective outcomes, suboptimal sample recruitment strategies, or insufficient consideration of bias. Although quality assessment was not an inclusion criterion for this systematic review, it was conducted to provide an overview of the quality of the eligible literature. Consequently, studies rated as “semivaluable” were still included in the data analysis. While these studies were limited in methodological rigor and offered less robust insights into the tools being evaluated, they contributed unique perspectives on health care workers’ trust in AI-CDSS tools that were not captured in other included studies. The included studies also discussed several limitations, including small sample sizes and various biases, such as potential selection bias, cognitive biases (eg, anchoring bias), and interviewer bias commonly associated with qualitative research. Other limitations included inaccuracies in self-reported data, regional differences in AI exposure, participants’ familiarity with the study context, and a focus on specific AI solutions or decision domains, all of which may limit the generalizability of findings.

**Table 1.** Critical Appraisal Skills Programme responses for each study included in the systematic review (n=23)^a^.

Study	1. Was there a clear statement of the aims of the research?	2. Is the qualitative methodology appropriate?	3. Was the research design appropriate to address the aims of the research?	4. Was the recruitment strategy appropriate to the aims of the research?	5. Were data collected in a way that addressed the research issue?	6. Has the relationship between the researcher and participants been adequately considered?	7. Have ethical issues been taken into consideration?	8. Was the data analysis sufficiently rigorous?	9. Is there a clear statement of findings?	10. How valuable is the research?
Jacobs et al [20]	Yes	Yes	Yes	Yes	Yes	Cannot tell	Yes	Yes	Cannot tell	Semivaluable
Wang et al [21]	Yes	Yes	No	Yes	Yes	Cannot tell	Yes	Cannot tell	Yes	Valuable
Micocci et al [22]	Yes	Yes	Yes	Yes	Yes	Yes	Yes	Yes	Yes	Valuable
Henry et al [3]	Yes	Yes	Yes	Yes	Yes	Yes	Yes	Yes	Yes	Valuable
Choudhury et al [23]	Yes	Yes	Yes	Yes	Yes	Yes	Yes	Yes	Yes	Valuable
Gunasekeran et al [24]	Yes	Yes	Yes	Yes	Yes	Yes	Yes	Cannot tell	Yes	Semivaluable
Choudhury [25]	Yes	Yes	Yes	Yes	Yes	Yes	Yes	Yes	Yes	Valuable
Ankolekar et al [26]	Yes	Yes	Yes	Yes	Yes	Yes	Yes	Yes	Yes	Valuable
Van Biesen et al [27]	Yes	Yes	Yes	Yes	Yes	Yes	Yes	Yes	Yes	Valuable
Sivaraman et al [28]	Yes	Yes	Yes	Yes	Yes	Yes	Yes	Cannot tell	Yes	Semivaluable
Amann et al [13]	Yes	Yes	Yes	Yes	Yes	Yes	Yes	Cannot tell	Yes	Valuable
Bach et al [29]	Yes	Yes	Yes	Yes	Yes	Yes	Yes	Cannot tell	Yes	Valuable
Burgess et al [30]	Yes	Yes	Yes	Yes	Yes	Yes	Yes	Yes	Yes	Valuable
Liu et al [31]	Yes	Yes	Yes	Yes	Yes	Yes	Yes	Yes	Yes	Valuable
Anjara et al [32]	Yes	Yes	Yes	Yes	Yes	Yes	Yes	Yes	Yes	Valuable
Jones et al [5]	Yes	Yes	Yes	Yes	Yes	Yes	Yes	Yes	Yes	Very valuable
Liu et al [33]	Yes	Yes	Cannot tell	Yes	Yes	Yes	Yes	Yes	Yes	Semivaluable
Chiang et al [12]	Yes	Yes	Yes	Yes	Yes	Yes	Yes	Yes	Yes	Valuable
Liaw et al [34]	Yes	Yes	Yes	Yes	Yes	Yes	Yes	Yes	Yes	Valuable
Nair et al [35]	Yes	Yes	Yes	Yes	Yes	Yes	Yes	Yes	Yes	Valuable
Yoon et al [7]	Yes	Yes	Yes	Yes	Yes	Yes	Yes	Yes	Yes	Valuable
Zheng et al [4]	Yes	Yes	Yes	Cannot tell	Yes	Yes	Yes	Cannot tell	Yes	Semivaluable
Vereschak et al [11]	Yes	Yes	Yes	Yes	Yes	Cannot tell	Yes	Cannot tell	Yes	Valuable

a Choudhury [36], Stacy et al [2], York et al [37], and Elareed et al [38] are cross-sectional quantitative studies not included in the Critical Appraisal Skills Programme analysis.

### Characteristics of Included Studies

Of the 27 included articles, summarized in Table 2 and Figure S1 in Multimedia Appendix 2, most were published recently: 12 (44%) in 2023, followed by 8 (30%) in 2022, 4 (15%) in 2024, and 3 (11%) in 2021. Geographically, most studies were conducted in the United States (n=12, 44%), followed by Europe (n=7, 26%), multinational collaborations (n=3, 11%), the United Kingdom (n=2, 7%), and 1 (4%) study each from China, Singapore, and Egypt. Most studies were conducted in hospital settings (n=17, 63%), across departments such as emergency care, radiology, and oncology, while 5 (19%) studies were conducted in primary care settings and 5 (19%) studies spanned both hospital and primary care environments. Study designs included qualitative research (n=16, 59%), mixed-methods studies (n=6, 22%), quantitative cross-sectional surveys (n=4, 15%), and 1 (4%) comparative evaluation study assessing AI-generated versus human-generated suggestions for clinical decision support. The study populations encompassed a wide range of health care providers such as physicians, nurses, nurse practitioners, general practitioners, intensive care unit clinicians, pharmacists, ophthalmologists, oncologists, interdisciplinary teams, behavioral health specialists, and AI practitioners. Sample sizes varied from small focus groups to cohorts exceeding 1000 individuals.

The included studies featured a wide range of AI-CDSS tools, demonstrating their application across various clinical functions and specialties (Table 2). These systems employ advanced technologies such as machine learning, deep learning, reinforcement learning, and explainable AI to support diagnostics, treatment planning, and clinical decision-making. Examples include the AI-based blood utilization calculator for improving transfusion procedures, the Brilliant Doctor system for dermatological diagnosis, machine learning and reinforcement learning models for providing sepsis treatment recommendations in intensive care unit settings, and QRhythm for identifying optimal rhythm management strategies in atrial fibrillation. Additional innovations are AI-CDSS tools for detecting and managing diabetic retinopathy, glaucoma, and cataracts; ChatGPT-enhanced electronic health record alerts for medication optimization in diabetes; as well as systems for lung cancer relapse prediction, vancomycin dosing, cardiovascular risk prediction, trauma radiography, and asthma management.

**Table 2.** Characteristics of included studies and evaluation of trust factors in AI^a^-CDSSs^b^ among health care workers, including study design, population, location, method of evaluating trust, along with a description of AI-CDSSs (n=27).

Study	Geography	Setting	Study design	Study population (number of participants)	Method of evaluating trust	Description of AI-CDSS	Evaluation of health care worker trust factor for AI-CDSS
Jacobs et al [20]	Multinational: the United Arab Emirates, Singapore, and Hong Kong	Hospital	Qualitative study	Physicians (n=9) and nurse practitioners (n=1) who are primary care providers	Semistructured qualitative interview	Machine learning models used to provide prognostic predictions and support treatment selection for major depressive disorder.	• Previous system utilization, including its use by other clinicians and validation through randomized controlled trials • Level of training received
Wang et al [21]	China	Primary care	Qualitative study	Clinicians with expertise in both Western and Traditional Chinese medicine (n=22)	Semistructured qualitative interview	A deep learning and knowledge graph–based AI-CDSS system (Brilliant Doctor).	• The “black-box” nature of the AI algorithm and its lack of transparency in the recommendations • Perceived threat to professional autonomy and decision-making, with the “click-through” approach disrupting workflows • Insufficient training on system features and functionality, along with clinicians’ understanding
Micocci et al [22]	United Kingdom	Primary care	Mixed-method study	General practitioners (n=50)	Semistructured qualitative interview	AI system developed to support the diagnosis of dermatological conditions.	• Accuracy of the AI system • General practitioners’ familiarity with AI • Previous experiences with similar technologies
Henry et al [3]	United States	Hospital	Qualitative study	Physicians (n=13) and nurses (n=7) worked at the emergency department, critical care, and general ward	Semistructured qualitative interview	A machine learning–based system called Targeted Real-time Early Warning System, designed to alert for sepsis detection, evaluate patients, and support treatment management.	• Direct experience with the system and observing its behavior over time • Endorsement and recommendations from colleagues and experts • Understanding the system’s development and validation process • Ability to customize the system and ask questions about its design
Choudhury et al [23]	United States	Hospital	Qualitative study	Clinicians involved in blood transfusion decision-making (n=10)	Semistructured qualitative interview	An AI-based blood utilization calculator designed to optimize blood transfusion practices.	• Workload • Usability • Impact on decision-making • Alignment with clinical judgment
Gunasekeran et al [24]	Multinational: more than 70 countries	Primary care and hospital	Mixed-method study	Ophthalmologists (n=1176)	Likert scales and dichotomous questions	Various AI-based assistive tools and clinical decision support applications used in ophthalmology to detect and manage eye diseases, including diabetic retinopathy, glaucoma, age-related macular degeneration, and cataract.	• Usability • Acceptable error levels and concerns over medical liability • Professional acceptance • Organizational support
Choudhury et al [25]	United States	Hospital	Mixed-method study	Clinicians who used the blood utilization calculator (n=119)	Semistructured qualitative interview	An AI-based blood utilization calculator.	• Perception of AI • Expectancy (effort and performance expectancy) • Perceived risk
Ankolekar et al [26]	The Netherlands	Hospital	Mixed-method study	Patients with non-small-cell lung cancer (n=257) treated at a single radiotherapy clinic, and lung cancer specialists (n=9)	Semistructured qualitative interview	CDSSs developed to support shared decision-making in lung cancer prognosis.	• Lack of external validation • Clinician experience • Perceived usefulness of CDSSs
Stacy et al [2]	United States	Hospital	Quantitative study	Health care workers involved included clinicians who manage patients with atrial fibrillation (n=33)	Likert scale (0‐5)	A 2-stage machine learning model–based tool, the QRhythm model, designed to identify the optimal rhythm management strategy.	• Accuracy of the AI recommendations • Transparency of the AI processes • Clinicians’ previous experiences with AI
Choudhury et al [36]	United States	Hospital	Quantitative study	Physician residents and fellows (n=111) and nurses (n=8)	Semistructured qualitative interview	An AI-based decision support system known as the blood utilization calculator.	• Perceived risk • Expectancy • Acceptance of the AI system
Van Biesen et al [27]	Belgium	Hospital	Qualitative study	Physicians (n=30)	Semistructured qualitative interview	AI-CDSS tools integrated into electronic health records.	• Transparency • Reliability • Perceived accuracy of the CDSSs
Sivaraman et al [28]	United States	Hospital	Mixed-method study	Intensive care unit clinicians (n=24)	Likert scale (0‐10)	A reinforcement learning model–based tool called the “AI Clinician,” designed to provide interpretable treatment recommendations for patients with sepsis in the intensive care unit.	• The credibility of the developers who created the AI-based tool • The perceived soundness of the methodology used to develop the tool
Amann et al [13]	Germany and Switzerland	Primary care	Qualitative study	Health care professionals, including physicians (n=7), occupational therapists (n=1), physiotherapists (n=4), neuropsychologists (n=2), stroke survivors (n=14), and family members (n=6)	Semistructured qualitative interview	AI-CDSS tools designed to act as administrative assistants for routine tasks and to aid in the diagnosis and treatment of complex stroke cases.	• Concerns that AI may lead to dehumanization in health care and erode patient-clinician trust
Bach et al [29]	Denmark	Hospital	Qualitative study	Ophthalmologists (n=7)	Semistructured qualitative interview	AI system for detecting diabetic retinopathy by analyzing color-coded assessments of fundus images and optical coherence tomography scans to determine the presence and severity of lesions.	• Accuracy and reliability of AI assessments, including its ability to minimize false positives/negatives • Failure of the AI system to detect severe abnormalities beyond its intended scope • Limitations in the AI system’s performance due to factors such as image quality
Burgess et al [30]	United States	Primary care and hospital	Qualitative study	Primary care provider (n=14), nurse practitioner/physician assistant (n=18), endocrinologist (n=5), pharmacist (n=2), and internal medicine (n=2)	Semistructured qualitative interview	A machine learning model trained on a large dataset of 141,625 patients with type 2 diabetes mellitus to optimize medication selection and predict the relative efficacy of different drug regimens in reducing hemoglobin A_1c_ levels.	• Comparison of AI-CDSS tools with the “gold standard” of randomized controlled trials in generating insights • Clinicians’ understanding of how the insights are generated and which outcomes the system is designed to optimize • Clinicians’ trust in the data, such as claims data, used to train the AI model
Liu et al [31]	United States	Hospital	Comparative evaluation	Clinicians (n=5)	Likert scale (0‐5)	ChatGPT, a large language model by OpenAI, used to improve CDSS alerts in electronic health records.	• Understanding • Relevance and clarity of AI suggestions • Usefulness • Acceptance • Workflow impact • Redundancy • Potential for bias
Anjara et al [32]	Spain	Hospital	Qualitative study	Oncologists with specialized training in treating lung cancer (n=10)	Think-aloud protocol	Explainable AI system based on a graph representation learning model for predicting lung cancer relapse.	• Perception of clarity • Credibility and utility • Information overload and the presence of example-based explanation • System’s alignment with clinical decision-making needs
Jones et al [5]	Multinational: Belgium, the United Kingdom, Italy, and China	Primary care and hospital	Qualitative study	Physician (n=24)	Semistructured qualitative interview	AI-powered CDSS used in the context of ophthalmology (ie, clinical care specializing in eye and vision health).	• Perception of clinicians’ control over decision-making • Medical errors • Legal responsibility/liability
Liu et al [33]	United States	Hospital	Qualitative study	Critical care pharmacists (n=13)	Semistructured qualitative interview	AI-CDSS tools designed to facilitate vancomycin dosing for hospitalized patients.	• Accuracy of recommendations • Rationale behind dosing • Transparency of the AI model • The black-box nature of AI recommendations • Complexity of algorithms
York et al [37]	United Kingdom	Hospital	Quantitative study	Clinicians with varying levels of training, including foundation year 1 (n=108), foundation year 2 (n=28), specialty trainee/core trainee 1‐2 (n=35), specialty trainee 3/specialty registrar or above (n=49), and medical students (n=77)	Semistructured qualitative interview	AI-CDSS tools applied in the development of skeletal radiography for trauma.	• Knowledge of AI • Confidence in interpreting radiographs • Level of training and experience of the clinician
Chiang et al [12]	United States	Primary care	Qualitative study	Ophthalmologists and optometrists from the University of California, San Diego (n=10)	Semistructured qualitative interview	AI-based decision support system designed to predict the risk of cardiovascular disease.	• Accuracy • Reliability • Usefulness
Liaw et al [34]	United States	Primary care and hospital	Mixed-method study	Physician (n=24)	Semistructured qualitative interview	Diabetes AI prediction tool designed to predict the risk of poor diabetes control.	• Accuracy of the tool • Transparency of the AI processes • Clinicians’ familiarity with AI
Nair et al [35]	Sweden	Primary care and hospital	Qualitative study	Physician (n=14); nurse practitioner/nurse/physician assistant (n=3); behavioral specialist (n=1); social worker(n=1); and other staff including front desk, administrative, or medical assistant (n=3)	Semistructured qualitative interview	AI-based decision support tool designed to reduce the risk of readmission in patients with heart failure.	• Stakeholder engagement • Perceived benefits • Transparency
Yoon et al [7]	Singapore	Hospital	Qualitative study	Clinicians (n=13) in 4 focus groups	Focus group discussion	AI-enabled prescription advisory tool.	• Interpretability of AI-generated recommendations • Transparency of the system • Clinicians’ previous experiences with AI
Zheng et al [4]	United States	Hospital	Qualitative study	Clinicians (n=14) who treated pediatric patients with asthma at 2 outpatient facilities	How-Might-We questions	Machine learning–based CDSS, the Asthma Guidance and Prediction System, for asthma management.	• Accuracy • Reliability • Explainability of the AI tool
Elareed et al [38]	Egypt	Hospital	Quantitative study	Physician (n=249)	Likert scale (0‐5)	General AI applications in health care, including potential uses in disease management and treatment.	• Job replacement by AI • Perceived usefulness • Reduction in workload • Impact on physician-patient relationship • AI to handle patient data responsibly
Vereschak et al [11]	France and Germany	Primary care	Qualitative study	AI practitioners, including bioengineers and researchers (n=1) and others (n=6), and AI decision participants, including a medical student (n=1) and others (n=6).	Semistructured qualitative interview	AI-assisted decision-making systems, particularly those employing machine learning techniques.	• AI transparency • AI literacy • Interpersonal relationships between stakeholders (developer and user) • The complexity of tasks

a AI: artificial intelligence.

b CDSS: clinical decision support system.

### Factors Influencing Health Care Workers’ Trust in AI-CDSS Tools

To analyze health care workers’ trust in AI-CDSS tools, we identified the methods used across 27 studies to assess trust-related elements. The majority employed semistructured qualitative interviews (n=19, 70%), followed by Likert scales (n=4, 15%), focus group discussions (n=1, 4%), How-Might-We questions (n=1, 4%), Likert scales combined with dichotomous questions (n=1, 4%), and the think-aloud protocol (n=1, 4%; Table 2). The assessment of trust in AI-CDSS tools revealed various factors that must be addressed to strengthen trust among health care workers (Table 2). Factors described in the study include experience with the AI system, colleagues’ recommendations, results from randomized controlled trials, and clinicians’ direct experience with the system over time. Transparency, accuracy, and the reliability of AI recommendations were identified as critical, with recurring concerns about the “black-box” nature of algorithms and the lack of clarity regarding how insights are generated. Additional factors influencing trust included perceived or actual risks, ease of use, organizational fit, and alignment with clinical judgment. Health care workers emphasized the importance of adequate training, customizable features, and the credibility of system developers. Trust perceptions were also shaped by considerations such as workload impact, acceptable error thresholds, and concerns around medical liability. Stakeholder involvement and familiarity with AI systems were described as contributing positively to trust in AI-CDSS tools. However, concerns about job displacement and the potential dehumanization of care emerged as significant challenges to fostering trust in these technologies.

### Insights Into Health Care Workers’ Trust in AI

**Figure 2.**
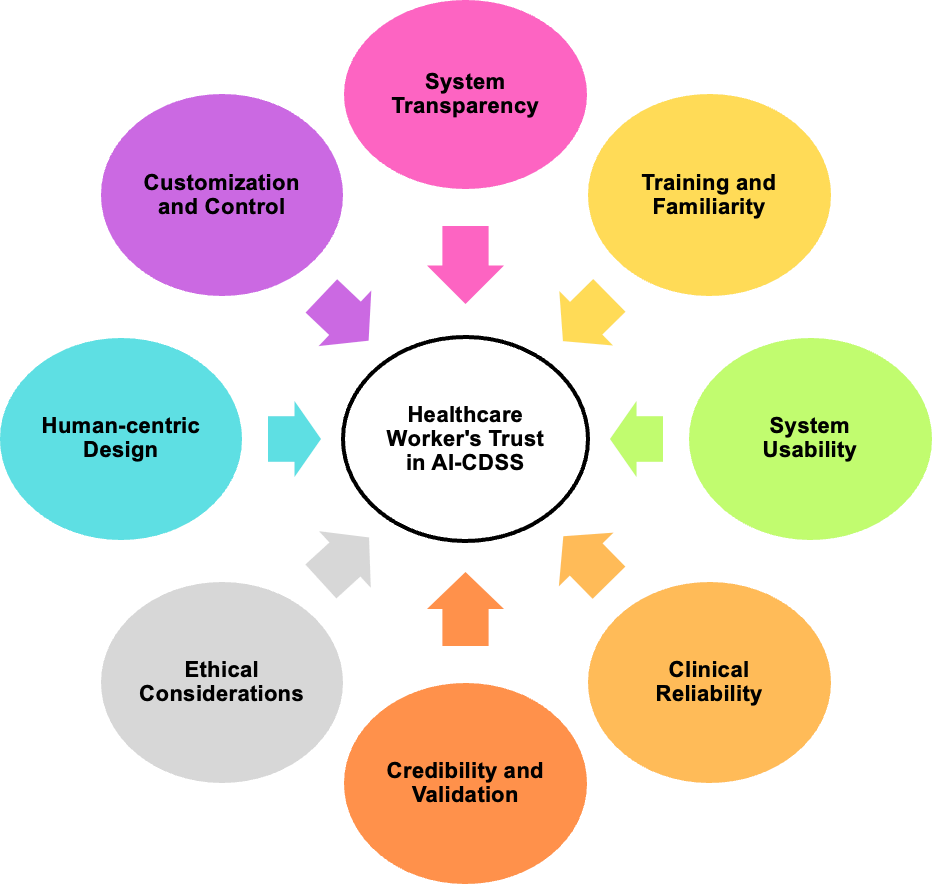
Overview of eight thematic areas related to healthcare workers’ trust in AI-based clinical decision support systems (AI-CDSS).

The synthesis of study findings on trust in AI-CDSSs revealed 8 key thematic insights (Table 3 and Figure 2). These include (1) system transparency, which emphasizes the need for clear and interpretable AI systems; (2) training and familiarity, which highlights the importance of educating and familiarizing health care workers with AI-CDSS; (3) system usability, which focuses on seamless integration into clinical workflows; (4) clinical reliability, which stresses the need for consistent and accurate system performance; (5) credibility and validation, which describe the importance of system validation across diverse clinical contexts; (6) ethical consideration, which examines issues such as medicolegal liability, fairness, and adherence to ethical standards ,(7) human centric design, which focus on piroitizing patient centered approaches in design and finally, (8) customization and control, which reflect the need for AI tools to adapt to specific clinical needs while ensuring health care providers retain decision-making autonomy.

These themes were explored through the enablers and barriers that influence health care workers’ trust in AI-CDSS. Among the enablers, prior system use and validation through randomized controlled trials were cited as key factors that boosted confidence in the AI systems. Familiarity and training with AI tools further strengthened clinicians’ trust, empowering them to make informed decisions. Additionally, observing the system’s performance over time and receiving endorsements from colleagues and domain experts contributed significantly to trust building. Furthermore, system usability, alignment with clinical judgment, and the ability to reduce workload emerged as important factors positively influencing trust. Transparency in the AI development process and the perceived credibility of the developers also played a critical role in fostering confidence. Finally, the explainability and interpretability of AI recommendations, along with the ability to customize the system and seek clarification, offered clinicians a greater sense of control, further enhancing trust.

However, the study revealed several barriers that erode trust in AI-CDSS. A major concern was the black-box nature of the AI algorithm, which renders recommendations opaque and difficult to interpret. Clinicians also expressed concerns about inadequate training, which diminishes their understanding and confidence in using these systems effectively. Additional barriers included workflow disruptions, perceived threats to professional autonomy, and doubts regarding the accuracy and reliability of AI-generated recommendations. Ethical considerations, such as fears of dehumanization in patient care and perceived risks of job replacement, added further complexity to trust in AI-CDSS. Concerns were also raised about the efficacy of these systems, particularly due to inadequate external validation and limited generalizability to diverse clinical contexts. Lastly, trust was further undermined by unresolved issues related to medical liability, potential algorithmic biases, and broader ethical risks.

**Table 3.** Eight thematic areas influencing health care workers’ trust in AI^a^-CDSS^b^, along with enablers, barriers, and recommendations (n=27).

Theme	Enablers	Barriers	Recommendations
System Transparency	• Prior system use and validation through randomized controlled trials	• Lack of transparency in AI algorithms (“black-box” nature) • Unclear recommendations	• Use interpretable algorithms • Provide clear, actionable recommendations
Training and Familiarity	• Training and experience with the AI system • Improved confidence and familiarity	• Insufficient training on system functionality	• Implement comprehensive training programs to build user confidence and understanding
System Usability	• Direct observation of system behavior • Endorsements from colleagues	• Workflow disruption • Perceived threat to professional autonomy (eg, “click-through” processes)	• Conduct hands-on training • Facilitate peer-led workshops to improve usability
Clinical Reliability	• Usability aligned with clinical judgment • Reduced workload	• Concerns about accuracy and reliability of AI recommendations	• Validate systems through randomized trials and real-world studies
Credibility and Validation	• Perceived robustness of AI development methods	• Limited external validation • Poor generalizability to diverse clinical settings	• Ensure external validation • Test across varied health care settings to build trust
Ethical Considerations	• Credibility of developers • Engagement with stakeholders	• Medical liability concerns • Fear of clinical errors	• Clarify legal responsibilities • Ensure strong validation to reduce liability risk
Human-Centric Design	• Explainable, interpretable AI recommendations	• Concerns about dehumanization of care • Threats to patient-clinician relationships	• Design AI to support (not replace) human judgment • Prioritize patient-centered care
Customization and Control	• Clinicians’ ability to customize the system • Ability to ask questions	• Perceived risks: bias, job displacement, and ethical concerns	• Involve stakeholders in the design process • Address ethical issues and bias transparently

a AI: artificial intelligence.

b CDSS: clinical decision support system.

## Discussion

### Principal Findings

The systematic review included 27 studies analyzing health care workers’ trust in AI-CDSSs. The article selection process began with 333 records and, after rigorous screening based on inclusion criteria, was narrowed to 27 studies. Most studies were recent (n=12, 44% from 2023) and conducted in hospital settings across diverse health care worker groups. Qualitative methods dominated (n=16, 59%), with sample sizes ranging from small focus groups to cohorts of over 1000 participants. The synthesis of findings highlights 8 thematic areas influencing health care workers’ trust in AI-CDSS tools, encompassing both enablers and barriers. Key enablers include prior system validation, transparency, training, usability, and alignment with clinical judgment. By contrast, barriers such as algorithmic opacity, inadequate training, workflow disruptions, and ethical concerns undermine trust. Based on these themes, we provide actionable recommendations for the design and implementation of AI systems that are more likely to be trusted and accepted by health care practitioners (Table 3).

### System Transparency

Fostering trust in AI-CDSSs among health care workers involves enhancing the transparency of AI algorithms and providing clear, practical, and actionable recommendations for clinical decision-making [39-41]. According to Nasarian et al [42], black-box models pose challenges in CDSS due to their limited interpretability, especially when compared with simpler white-box models that offer transparent results without requiring additional parameters. While black-box models often deliver high accuracy, their opacity can lead to confusion about how decisions are made—and, in some cases, may result in overreliance on the system, particularly among less experienced clinicians who may lack the expertise to interpret AI outputs effectively [22]. Gray-box models, positioned between the extremes of black-box and white-box models, offer a balance between complexity and interpretability, provided they are designed effectively [42]. Interpretability should be incorporated throughout the entire process, from data preprocessing and model selection to postmodeling phases. However, most existing AI-CDSS tools focus primarily on postmodeling explainability. To reduce skepticism surrounding the “black-box” nature of AI systems, developers should ensure transparency in the rationale behind recommendations at every stage of the development pipeline [42].

### Training and Familiarity

To improve trust in AI-CDSS, comprehensive training programs on AI tools for health care workers play a vital role [39,43]. These programs not only build familiarity with the systems but also enhance users’ confidence in their reliability and functionality. Dlugatch et al [6] discussed the impact of AI on health care workers. As AI technology begins to surpass human capabilities, the epistemic authority of medical practitioners risks being undermined, challenged, or even supplanted [6]. This may lead health care professionals to view AI-CDSS tools as replacements rather than assistants. To address these challenges, training programs should educate health care workers on how AI systems are developed, including their capabilities, limitations, and potential pitfalls.

### System Usability

To improve system usability and alignment with clinical judgment, hands-on training and peer-led workshops should be conducted [39,43]. These approaches not only enhance health care workers’ understanding of AI systems but also improve their practical usability. According to Task Technology-Fit (TTF) theory, users are more likely to adopt a technology only if it aligns with their tasks and improves performance. However, external influences and uncertainty surrounding AI can introduce biases that either encourage or deter clinicians from adopting such technologies in the future [25]. This highlight the importance of peer-to-peer sharing of experiences with AI-CDSS tools.

### Clinical Reliability

To ensure clinical reliability, AI systems should demonstrate accuracy and consistency through real-world testing and randomized controlled trials [44-46]. Micocci et al [22] noted that AI systems, like human clinicians, are inherently imperfect and should be designed to complement, not replace, the clinician’s holistic understanding of each clinical scenario. While AI can offer valuable decision support, the ultimate responsibility for diagnostic resilience lies with the clinician, who retains the authority to accept or reject AI recommendations [22].

### Credibility and Validation

Trust in AI-CDSS can be further fostered through external validation of the system in diverse clinical settings, which can help demonstrate the soundness of the AI methodology used in its development [47-49]. Nair et al [35] mentioned that clinicians express fatigue from the integration of AI-based tools into workflows, especially when organizations are reluctant to discontinue ineffective technologies. This underscores the crucial role of tool developers in thoughtfully managing and thoroughly validating systems across varied contexts to avoid adding further burden to health care workers.

### Human-Centric Design

The importance of human-centric design cannot be overstated in fostering trust in AI-CDSS [50,51,52,53,54, 55,56 ]. Amann et al [13] raised concerns about technology-induced dehumanization in patient care and its impact on the patient-clinician relationship. Sivaraman et al [28] and Jacobs et al [5] discussed the important role of a sociotechnical lens in designing AI-CDSS, emphasizing the need to integrate environmental and social factors into system development. Furthermore, Alruwaili et al [8] discussed that health care professionals, such as nurses, have varying concerns about AI’s impact on the human aspect of care, while others recognize its potential benefits. This highlights the importance of incorporating humanistic elements in the design of AI-CDSS as supportive tools that enhance, rather than detract from, patient care.

### Ethical Concerns and Guidelines

Clear guidelines on roles and responsibilities, along with robust validation of AI tools, will address liability related to ethical concerns, which will help alleviate concerns and build trust [57-61 ]. Gunasekeran et al [24] and Jones et al [5] noted that health care workers fear the medicolegal impact of AI-CDSS systems. Providing explicit guidance on the capabilities of AI-CDSS and clearly delineating the roles and responsibilities of health care workers can further help mitigate concerns related to medical errors and liability. Ethical AI frameworks, such as the European Commission’s Ethics Guidelines for Trustworthy AI, the EU AI Act, and the OECD’s AI Ethics Guidelines, offer specific guidance for the development of AI-CDSS in the health care sector [62-64]. These frameworks not only help reduce ethical concerns but also promote human-centric design, which can enhance health care workers’ trust in AI-CDSS.

### Customization and Control

Trust in AI-CDSS can be fostered through collaboration, coordination, and meaningful stakeholder engagement during system design, helping to eliminate ethical concerns and fears of job replacement among health care workers [65-72]. Chiang et al [12] emphasized the importance of securing support from a variety of stakeholders, such as organizational leadership and end users, early in the development process to improve trust in AI-based tools. Ball et al [73] also highlighted the role of collaboration and continuous communication through a “human-in-the-loop” approach, which integrates human expertise and addresses the limitations of AI algorithms. Involving direct end users, such as health care workers, during the development phase enables them to better understand the supportive role of AI-CDSS, rather than perceiving it as a threat to their jobs. Furthermore, engaging a range of stakeholders can help reduce ethical concerns by raising possible issues, such as harm to patients, early in the process. This, in turn, allows developers to make necessary modifications and improve trust in the implementation of AI-CDSS tools [11].

### Limitations of the Study

This systematic review has certain limitations. First, it included only studies published in English and did not account for AI system studies from nonindexed journals, which may limit the relevance of the findings to non–English-speaking or unpublished research. Second, the quality assessment was conducted using the CASP checklist, which evaluates only the qualitative elements of included studies, regardless of their overall design. This may have limited the generalizability of findings derived from nonqualitative studies. Third, although we aimed to conduct a meta-analysis, including a subanalysis or comparative analysis, we were unable to do so due to the high level of heterogeneity across studies and the lack of detailed demographic information. Additionally, sufficient data on health care roles and geographic contexts associated with the qualitative quotes and outcomes hindered our ability to conduct a comparative analysis. Lastly, the study was formative in nature, with categories and components generated through a subjective synthesis process, which may introduce interpretive bias. Despite these limitations, the synthesis and recommendations from this study help bridge existing gaps and provide specific themes to foster health care workers’ trust in AI-CDSS. Future studies should consider incorporating more diverse demographic data, performing cross-cultural studies, and exploring contextual differences in trust across various health care professional groups to address these gaps more comprehensively.

### Conclusions

Our systematic review of 27 studies identifies 8 key themes influencing health care workers’ trust in AI-CDSS tools. We highlight important enabling factors such as transparency, training, usability, clinical reliability, and alignment with clinical judgment. Conversely, barriers include algorithmic obscurity, inadequate training, and ethical concerns. Based on these findings, we recommend prioritizing the development of transparent AI models, implementing comprehensive training initiatives, and conducting practical workshops with real-life testing to foster sustained trust in AI-CDSS among health care workers. Moreover, integrating human-centered design and addressing ethical considerations are crucial to ensuring that AI tools enhance, rather than hinder, the patient-health care worker relationship. Despite limitations, such as the exclusion of non-English studies, heterogeneity in study designs, and a lack of detailed data, the analysis is limited in conducting further exploration. Nevertheless, it bridges existing gaps and provides specific themes to foster the trust of health care workers in AI-CDSS. The identified thematic areas, along with our recommendations, establish a foundation for forthcoming research and development of AI-based tools to ensure that AI-CDSS are efficient, reliable, and trustworthy for health care workers.

## Supplementary material

10.2196/69678 Multimedia Appendix 1 Search strategies.

10.2196/69678 Multimedia Appendix 2 Additional analysis.

10.2196/69678 Checklist 1 PRISMA (Preferred Reporting Items for Systematic Reviews and Meta-Analyses) checklist.

## Abbreviations

AI: artificial intelligence
CASP: Critical Appraisal Skills Programme
CDSS: clinical decision support system
MeSH: Medical Subject Headings
PICO: population, intervention, comparison, and outcome
PRISMA: Preferred Reporting Items for Systematic Reviews and Meta-Analyses
