# Supplementary material for: Trust in Artificial Intelligence–Based Clinical Decision Support Systems Among Health Care Workers: Systematic Review
Source: J Med Internet Res. 2025 Jul 29;27:e69678. doi: 10.2196/69678 (PMC12440830; doi:10.2196/69678)
Supplement: Multimedia Appendix 1 [file jmir-v27-e69678-s001.docx]

## **Appendix 1 - Search strategies detail**

This appendix outlines the detailed search strategies, including medical subject headings (MeSH) terms, free-text keywords and Boolean operators used each database search.

**PubMed**

The following search strategy was employed in PubMed to ensure comprehensive coverage:

(("trust" OR "acceptance" OR "perception") AND ("artificial intelligence" OR "AI") AND ("decision support systems" OR "clinical decision support" OR "AI-based decision support")) AND ("healthcare workers" OR "clinicians" OR "medical professionals" OR "healthcare providers") AND ("2020"[Date - Publication] : "2024"[Date - Publication])

**Scopus**

The search was conducted using TITLE-ABS-KEY fields to retrieved relevant content:

TITLE-ABS-KEY ( "trust" OR "acceptance" OR "perception" ) AND TITLE-ABS-KEY ( "artificial intelligence" OR "AI" ) AND TITLE-ABS-KEY ("decision support systems" OR "clinical decision support" OR "AI-based decision support" ) AND TITLE-ABS-KEY ( "healthcare workers" OR "clinicians" OR "medical professionals" OR "healthcare providers" ) ) AND PUBYEAR > 2019 AND PUBYEAR < 2025

**Google Search**

The following free search terms was used to identify relevant studies:

("trust" OR "acceptance" OR "perception") AND ("artificial intelligence" OR "AI") AND ("decision support systems" OR "clinical decision support" OR "AI-based decision support") AND ("healthcare workers" OR "clinicians" OR "medical professionals" OR "healthcare providers") AND (2020..2024)
