# Supplementary material for: Trust in Artificial Intelligence–Based Clinical Decision Support Systems Among Health Care Workers: Systematic Review
Source: J Med Internet Res. 2025 Jul 29;27:e69678. doi: 10.2196/69678 (PMC12440830; doi:10.2196/69678)
Supplement: Multimedia Appendix 2 [file jmir-v27-e69678-s002.docx]

**Supplementary materials**

|  |  |
| --- | --- |
|  |  |

Figure S1: Characteristics of Included Studies, Including Published Year, Locations, and Study Design

(n = 27)

|  |
| --- |

Figure S2: Method of assessing trust in included studies (n=27)

Table S2: Mixed Methods Appraisal Tool (MMAT) of included studies (n=27)

| Study ID | **SCREENING QUESTIONS** | | **3. NON-RANDOMIZED STUDIES** | | | | | |
| --- | --- | --- | --- | --- | --- | --- | --- | --- |
|  | S1. Are there clear research questions? | S2. Do the collected data allow to address the research questions? | 3.1. Are the participants representative of the target population? | | 3.2. Are measurements appropriate regarding both the outcome and intervention (or exposure)? | 3.3. Are there complete outcome data? | 3.4. Are the confounders accounted for in the design and analysis? | 3.5. During the study period, is the intervention administered (or exposure occurred) as intended? |
| Jacobs et al.2021[20] | Yes | Yes | Yes | Yes | | Yes | Yes | Can't Tell |
| Wang et al.2021[21] | Yes | Yes | Yes | Yes | | Yes | Yes | Yes |
| Micocci et al.2021[22] | Yes | Yes | Yes | Yes | | Yes | Yes | Yes |
| Henry et al.2022[3] | Yes | Yes | Yes | Yes | | Yes | Yes | Yes |
| Choudhury et al.2022[23] | Yes | Yes | Yes | Yes | | Yes | Yes | Yes |
| Gunasekeran et al.2022[24] | Yes | Yes | Yes | Can't Tell | | Yes | Yes | Can't Tell |
| Choudhury et al.2022[25] | Yes | Yes | Yes | Yes | | Yes | Yes | Yes |
| Ankolekar et al.2022[26] | Yes | Yes | Yes | Yes | | Yes | Yes | Yes |
| Stacy et al. 2022 [2] | Yes | Yes | Yes | Yes | | Yes | Yes | Yes |
| Choudhury et al.2022[27] | Yes | Yes | Yes | Yes | | Yes | Yes | Yes |
| Van Biesen et al.2022[28] | Yes | Yes | Yes | Yes | | Yes | Yes | Yes |
| Sivaraman et al.2023[29] | Yes | Yes | Yes | Yes | | Yes | Yes | Can't Tell |
| Amann et al.2023[13] | Yes | Yes | Yes | Yes | | Yes | Yes | Yes |
| Bach et al.2023[30] | Yes | Yes | Yes | Yes | | Yes | Yes | Yes |
| Burgess et al.2023[31] | Yes | Yes | Yes | Yes | | Yes | Yes | Yes |
| Liu et al.2023[32] | Yes | Yes | Yes | Yes | | Yes | Yes | Yes |
| Anjara et al.2023[33] | Yes | Yes | Yes | Yes | | Yes | Yes | Yes |
| Jones et al.2023[5] | Yes | Yes | Yes | Yes | | Yes | Can't Tell | Yes |
| Liu et al.2023[34] | Yes | Yes | Yes | Yes | | Yes | Yes | Yes |
| York et al.2023[35] | Yes | Yes | Yes | Yes | | Yes | Yes | Yes |
| Chiang et al.2023[12] | Yes | Yes | Yes | Yes | | Yes | Yes | Yes |
| Liaw et al.2023[36] | Yes | Yes | Yes | Yes | | Yes | Yes | Yes |
| Nair et al.2023[37] | Yes | Yes | Yes | Yes | | Yes | Yes | Yes |
| Yoon et al.2024[7] | Yes | Yes | Yes | Yes | | Yes | Can't Tell | Yes |
| Zheng et al.2024[4] | Yes | Yes | Yes | Yes | | Yes | Yes | Yes |
| Elareed et al.2024[38] | Yes | Yes | Yes | Yes | | Yes | Yes | Yes |
| Vereschak et al.2024[11] | Yes | Yes | Yes | Yes | | Yes | Yes | Yes |

Table S3 Outcomes of Included Studies with Supporting Quotes along Quantitative Results Related to Healthcare Workers’ Trust in AI-Based Clinical Decision Support Systems (AI-CDSS) (n = 27).

| Study ID | Outcome related to trust in AI based CDSS | Quotes / Quantitative result |
| --- | --- | --- |
| Jacobs et al.2021[20] | - Decision support tools (DSTs) need to account for the broader healthcare sociotechnical system, including clinical processes, patient preferences, resource constraints, and existing domain knowledge. - Current trends in explainable AI may be inappropriate for clinical environments, and new approaches are needed to design DSTs for real-world medical systems. | - “I think the biggest thing is just getting behind how you validated your data, how you validated your model ...I don’t know if you necessarily need to get into super nitty-gritty details” - P6 - “If a major medical society is sort of putting this forth,my colleagues are using it, and I hear people saying that it’s that it works, then I am comfortable with it.” - P7 - “If you could show that patients have a better response to treatment by use of the algorithm, that would be amazing. If you can show that patients actually are more likely to adhere to treatment, that would be important as well, or that patients are less likely to develop adverse side effects that leads to stopping medications. It would be nice to do a trial with outcomes like that.” - P9 |
| Wang et al.2021[21] | - The study found that the AI-CDSS system faced various challenges in being adopted by clinicians in the rural Chinese context, including misalignment with the local workflow and context, technical limitations and usability barriers, and issues related to transparency and trustworthiness of the AI system. | - “The medicine description provided by AI-CDSS, such as how much to take, is not always accurate. Take’tamsulosin’ as an example, it is used to treat prostate issues. AI-CDSS says take one pill per day so Ifollowed its guideline. But some older adults have very serious prostate problem, just taking one pill isnot effective. Some of them decided to take two pills per day without consulting us, but they said it workswell. Therefore, I went down to the pharmacy and checked the description of this medicine, and foundthat it says take one to two pills per day instead of strictly taking one.” - "Doctors and patients are friends, we usually have a good relationship. It is possible that the prescriptionwe gave to the patient is not working. I’ll just recommend them to go to higher-tier hospitals for furtherexamination. They understand us too, it is not like I intentionally gave you a wrong medicine or made youto be uncomfortable. [...] But if the AI system [directly] gives him a prescription that is not working, orunfortunately it causes some adverse events. The patient must complain about it. And more importantly,there is an accountability issue in there. Who is responsible for that?”" |
| Micocci et al.2021[22] | - AI has the potential to assist GPs, building trust through transparency and education is crucial for its successful integration. | - When the AI provided erroneous information, only 10% of the GPs were able to correctly disagree with the indication of the AI in terms of diagnosis (d-AIW M: 0.12, SD: 0.37), and only 14% of participants were able to correctly decide the management plan despite the AI insights (d-AIW M:0.12, SD: 0.32) |
| Henry et al.2022[3] | - Clinicians did not view the ML-based system as a replacement for their clinical judgment, but rather as a partner that augmented their diagnostic and treatment management processes. - Clinicians developed trust in the ML-based system through a variety of mechanisms, including direct experience, expert endorsement, and customization of the system to their needs. - Some barriers to the use of ML in medicine remain, such as concerns about over-reliance on automated systems and the potential for standardization of care. | - “I think we try to get them in front of a provider a little bit quicker or get some of the stuff started out in triage.” - “For clinicians, I think just understanding [that] this is a machine learning tool and it does data mining, I think will be more than enough.” - “I’d want to understand the population it was derived from… and then I’d want to see the population that they validated it on afterwards…whether that group looks like the patients that I’m treating.” - “I need to understand the motivation behind that tool because when I apply that tool, I’m applying the judgment of the creators of that tool.” - “I think [that] there are a lot of people, frankly, that will quickly default to having a tool tell them what to do and stop assessing, and I hope that’s not true, but I’ve seen it happen.” |
| Choudhury et al.2022[23] | - BUC was beneficial for standard care patients but posed usability challenges in complex cases, highlighting the importance of a user-centered design | - “I think it’s helpful because it explains like hemoglobin of several patients. If a patient has low platelets, you might have a higher hemoglobin goal. Um, so it’s nice to have that spelled out for you, so you don’t have to look it up elsewhere and then come back and make the decisions.” - “I like having the guidelines built-in so that you know when you’re doing something that is, um, the, that is the guideline or evidence based. And, you know, when you are deviating from that and therefore hopefully have a good reason for it and are at least cognizant of the fact that you're deviating.” - “If BUC is telling me that I’m ordering too much blood, I go back, thinking, okay, does the patient need this much blood? So, it’s more like I’m ensuring I follow the standard of care, except for those exceptional patient circumstances.” |
| Gunasekeran et al.2022[24] | - AI has strong potential as an assistive tool in ophthalmology, but additional support for organizational adoption and training is recommended to address barriers. COVID-19 pandemic was found to catalyze interest in AI adoption due to reduced provider-patient contact and enhanced screening needs. | - Many participants indicated that they strongly agree or agree that clinical AI will facilitate improvements in accessibility (84.7%, n = 785/927), affordability (61.9%, n = 574/927), and quality (69.4%, n = 643/927) in eye care services |
| Choudhury et al.2022[25] | - Emphasized the benefits of AI technology and addressing risk perceptions can improve clinicians' intent to use AI-based systems. | - “the greatest challenge to AI in these healthcare domains is not whether the technologies will be capable enough to be useful, but rather ensuring their adoption in daily clinical practice” |
| Ankolekar et al.2022[26] | - CDSSs have the potential to support shared decision-making in lung cancer treatment, but require external validation and integration into clinical practice. | - ‘[Models] must naturally be validated on large groups, and clinical factors must be considered. And even then, there is still a large variation in a result of such a model. So yes, it still remains difficult’. (Clinician 8) |
| Stacy et al. 2022 [2] | - Enhancing transparency and providing education about AI systems can improve trust among healthcare professionals. | - Trust in the app similarly varied. To the prompt “I trust the recommendations provided by the QRhythm app,” 1 provider (17%) somewhat disagreed, 2 (33%) were neutral, and 3 (50%) somewhat agreed |
| Choudhury et al.2022[27] | - The mediating effect of trust (the direct negative association between ‘risk perception’ and ‘trust,’ the direct positive association between ‘trust’ and ‘intent to use’ BUC, and indirect negative association of ‘risk perception’ and ‘intent to use’ BUC) imply that increasing trust in BUC, in general, can result in low-risk perception and high willingness to use the system, both of which are potential precursors to ‘automation bias’. | - Clinicians had moderately high ‘trust’ in BUC with a mean of 5.64. Clinicians also perceived the BUC as low risk, with a mean of 1.89 out of 5. Expectancy and intention to use BUC ranged from neutral to moderately high, with means of 3.62 and 3.50, respectively. "Overall, I trust the BUC." – (T1) [min 1-max 7,mean 5.66, Sd 0.92] “I trust the information I receive from the BUC.” – (T2) [min 1-max 7,mean 5.63, Sd 0.92] |
| Van Biesen et al.2022[28] | - The correctness of its advice absolute truth and certainty are rare in medical conditions. Therefore, it is essentialthat a CDSS can express this uncertainty in itsadvice. - CDSS can produce advice on request, but also in anunsolicited (automated) fashion while working with the system. This can interrupt the workflow, meaningthe user is distracted from her activity and needsto perform an unplanned action. | - “Not only the system, but also medicine [as a fieldof study] has to have a certain level of accuracy [inorder for these AI to function properly]. ” (R1) - “the quality and performance should be tested in arandomized trial” (R13) - “these (CDSS) should be peer reviewed, how elsewould I know their performance? ” (R12) - “if we start using them (CDSS), our confidence willgrow as we will better understand what triggers thesystem and what makes it go astray” (R3) - “In medicine it is always importantto doubt. … Our domain [medicine] is very hard toautomate, because it is difficult to reduce it to welldefinedpatterns. With us there are way too manydimensions to take into account.” (R23). - “[Unlike with medical decisions] I do trust the AI when it takes administrative decisions. Those do not look difficult to me. ” (R14). - “[Administrative tasks] are trivial. They are very easy and should, obviously, be integrated [in the system]. (R6) |
| Sivaraman et al.2023[29] | - Providing clinicians with explanations of AI recommendations increased their perceived usefulness of the AI and confidence in their own decisions. - Clinicians engaged in a nuanced process of selectively incorporating aspects of the AI's recommendations into their decision-making, rather than simply accepting or rejecting the recommendations. - AI systems could be designed to better support clinicians' negotiation of recommendations by highlighting the most important aspects rather than providing a single, rigid recommendation. | - “I would not have guessed that the decision or the recommendation was being based on something like a BUN [blood urea nitrogen] change. I assumed it was based on the CVP [central venous pressure], and I don’t think that CVP was considered in [the Feature Explanation chart]. And so it kind of makes you try and guess where the recommendations are coming from, and you spend a little bit more mental energy thinking about that.” - AI usefulness - (F (2,69) = 4.251, p = 0.03), text only condition ( delta =0.83 , 95% CI [0.24,1.43],p = 0.018), alteranative treatment(delta = 0.75,95%CI [-0.03,1.53],p=0.12) |
| Amann et al.2023[13] | - Participants envisioned a range of potential roles for medical AI in stroke care, from administrative assistant to fully autonomous system - While participants were generally positive about the potential benefits of medical AI, they also cautioned against viewing it as a panacea that will solve all healthcare problems. - Participants emphasized the importance of relational aspects in healthcare and expressed concerns that medical AI could negatively impact the doctor-patient relationship. | - “I think this [medical AI] is good, because it means that data can be collected again for the future, which can make any programs more precise, which can set the focus more precisely for an evaluation and then a recommendation as to what is good for the individual in order to get well again [after suffering a stroke].” (Pat8) - "I just hope from something like that, so from computer programs or algorithms actually, that assumptions that we, I think, always make in everyday life as humans—because someone is old or somehow looks like that or is old on paper—will be less incorporated [in the decisionmaking process], so this subjectivity." (HCP13) - “I would rather assume that the problem is not that you have the wrong options [provided by the AI system], but rather that you generally lack the resources to properly implement the options that are available. So, for example, sufficient physiotherapy in the outpatient area or something like that. That a computer-aided decision or simulation of different options would not change anything about the problem that already exists. would not change the problem that already exists.” (HCP4) - “Well, one shouldn’t overestimate AI, I have a feeling. It’s not the solution.. [. . .] Nobody is thinking, should we really do this? Do we need to do that? And what are the long-term consequences? And that’s where I think we tend to go too far, especially in healthcare, and by [introducing] potential solutions or improvements often we create new problems, which you can’t really anticipate.” (HCP0) - “I can imagine that there is a danger that health professionals will rely more on artificial intelligence and perhaps fixate on it and pay less attention to the patients and their wishes.”(HCP8) |
| Bach et al.2023[30] | - Ophthalmologists were aware of cognitive biases like anchoring bias when using AI-powered decision support systems, but were concerned about the impact of bias mitigation strategies on workflow efficiency. - Ophthalmologists had mixed expectations about the potential benefits of bias mitigation strategies on diagnostic accuracy, with some believing their accuracy could not be further enhanced and others seeing potential benefits, especially for less experienced clinicians. - Ophthalmologists expressed a desire for more capable AI systems that could detect a wider range of abnormalities, rather than just microaneurysms and haemorrhages, in order to be more open to bias mitigation techniques. | - “The AI system does not performwell enough for me to ignore the green images”. P1 (ophthalmologists) - there if all the images are green” (P5), and “I look through all of the images, and if it [the AI system] says they are all green, well then I go through the images slightly faster” (P5). Both P1 and P4 expressed a similar sentiment, with P4 specifically pointing to an increased sense of confidence when she agreed with the AI system: - “[the green labels] just give me a feeling of security” - “the colours do not matter, unless it’s all green, in which case I go through them quickly” - “If I have some that are yellow or red—and it really doesn’t matter whether they are one or the other—then I look at them very carefully”. |
| Burgess et al.2023[31] | - The paper provides a set of 6 design principles for developing effective AI-supported CDS systems.  1. Account for what is possible and realistic for the patient and the clinical context. Algorithms that over-optimize disease outcome metrics can lead to unrealistic insights. 2. Give the clinician the ability to weigh patient-specific factors that cannot be easily inferred automatically; give the clinician agency/control over model output. 3. Do not introduce "research" tasks for clinicians into patient visit workflow. 4. The introduction of the AI tool is a core opportunity for trust building. 5. Create networked systems designed for collaborative use by patients and healthcare staff throughout the patient’s care pathways. 6. Pinpoint where complex decisions need to take place in a clinical workflow versus tools that provide blanket data that physicians already know. | - “If you could show that patients have a better response to treatment by use of the algorithm, that would be amazing. If you can show that patients actually are more likely to adhere to treatment, that would be important as well, or that patients are less likely to develop adverse side effects that leads to stopping medications. It would be nice to do a trial with outcomes like that.” - P9 - “So truthfully, I would take a step back because it’s not that common that nortriptyline is a medication I think about as a first or even a second or third line agent, unless they have other conditions that I know [tricyclic antidepressants] can treat. So I would really take a step back and think about the patient’s pain. Do they have really bad migraines, that I think will get significant benefit from the TCAs. It would definitely give me pause if that was the most favorable medication to come up as a suggestion on this.” – P11 |
| Liu et al.2023[32] | - AI-generated suggestions can complement human efforts in optimizing CDS but should be refined for greater acceptance and workflow integration | - AI generated suggestions received lower scores for usefulness (AI:2.761.4, human: 3.561.3, P<.001) and acceptance (AI: 1.861,human: 2.861.3, P<.001). The overall scores were human:3.660.6 and AI: 3.360.5 (P<.001). |
| Anjara et al.2023[33] | - The study recommends improving the explanation model and including context such as cohort size and accuracy metrics to build clinician trust | - "“I would like someone to explain it to me more. I know this is very difficult so I won’t understand all the AI explanations. . .[but] I would want someone to explain the method more.” - “Here everything is clearer, there’s more data about the patients. It expresses pretty well the differences between them and what they have in common. It seems pretty clear, it’s quite visual. It seems simple.” - “This is more useful for research or for comparing patients but in our daily work it doesn’t provide much information. It provides information to compare patients, it’s more general,not for individual patients. To compare one, two or three patients it’s not very relevant clinically speaking. The example is quite clear but it’s not very relevant in our daily work, to tell you the truth.”" |
| Jones et al.2023[5] | - Understanding the nuanced meanings of trust and trustworthiness is essential for advancing the debate on AI in healthcare | - [T]there cannot be two right answers to the question of how a patient should be diagnosed, as might be the case in a “negligent treatment” case. Rather the diagnosis is simply wrong, and an expert witness who claims that a pathologist would have acted competently by missing obvious signs of melanoma was not expressing a defensible opinion. |
| Liu et al.2023[34] | - To enhance compliance with AI recommendations, improve transparency and integration of AI into clinical workflows. - The study emphasizes the need for trust in AI tools to improve clinical decision-making and highlights the importance of understanding clinician perceptions for successful AI integration. | - “You know, if I was very different from the AI, I would double-check myself. I would take it out and takeit seriously, and maybe I missed something. So I think it would be beneficial even though I may notagree with its dose.” - “I think if in this case, it’s the AI suggested that there was a 70% probability that I was overdosing;otherwise, I won’t change my dosage. In short, I don’t fully trust it” - “I would change the dose because I was on the higher side and would want to minimize renal injury. It’sslightly lower than my dose. I prefer the AI’s recommendation and would have chosen that” - “Oh, I think I would follow my dose. I don’t think that trained model recommendation because I’ve given hisage and his body weight and is severity illness, I would want to be a little more aggressive.” - So expected half-life for the patient, what the peak value would be, what the trough value would be, what area under the curve would be within AI’s scheme compared to our scheme. And think that those would both be helpful tools as well to give us some sort of objective sense that my dosing really not appropriate here.” |
| York et al.2023[35] | - There is clear support for the development of AI systems in healthcare, particularly in skeletal radiography, and efforts should be made to improve education on AI among clinicians. | - Participants indicated substantial favourability towards AI in healthcare (7.87) and in AI applied to skeletal radiography (7.75). There was a preference for a hypothetical system indicating positive findings rather than ruling as negative (7.26 vs 6.20). |
| Chiang et al.2023[12] | - The major conclusion of the study is that healthcare workers have varying levels of trust in AI-based DSSs, and that improving the transparency and explainability of these systems could help increase trust. | - There are two key findings in our study: 1) clinician perceptions were somewhat positive towards the trustworthiness and utility of AI-predicted VF metric, and 2) clinicians were less likely to use the AI output in their decision making as glaucoma severity increased. Overall, the mean Likert scale score for trustworthiness and utility of the predicted MD were 3.27 and 3.42 respectively |
| Liaw et al.2023[36] | - Enhancing transparency and providing education about AI systems can improve trust among healthcare professionals. | - Would it make care worse? Yeah, potentially...So ifyou’re prompted to prescribe medications...for peoplewho are not yet at a certain level of risk, the [benefitto harm] ratio becomes smaller. [Physician, academichealth center] - Racial bias is...something that’s implicitly existent innormal data sets...this is something that justcompounds...It’s like a small mistake that compoundsinto something bigger. [Physician, private solo orgroup practice] - If it’s things that are [inaccurate and] manuallyentered into the EHR system that are driving this...,it certainly could create false alerts and waste timeor...miss people who actually are at riskbecause...things weren’t...entered correctly, or leftblank. [Physician, private solo or group practice] - It’s only useful if I trust the information. [Physician,academic health center] - ...you could apply the same sort of thing to preventivecare to any chronic disease to including depression,hypertension, coronary disease. [Physician, academichealth center]...how likely is this person going to follow through ontheir screenings, [like] getting their mammogram?[Physician, private solo or group practice] |
| Nair et al.2023[37] | - Fostering trust through transparency and stakeholder engagement is crucial for the successful implementation of AI in healthcare. | - We reduce enormous suffering. We make their qualityof life better at home, AND we can get an economiclift in our region. Every one of the days is expensive.If we see that it is a patient with high risk, we can prioritize a visit to the HF clinic instead of sending the remittance to primary care. That should also allow for a quicker management. |
| Yoon et al.2024[7] | - Enhancing transparency and understanding of AI tools is crucial for building trust among clinicians | - When it [APA] was launched, a lot of us were notvery sure how it was developed. I think part of thereason why we did not use it very much is alsobecause we are not so familiar with how this systemcame about, what kind of information was used, andwhere the information came from. Is it also possiblethat critical information was not captured in thesystem? I can't trust totally, and [I am] not confidentwith what I'm seeing at the moment. [FGD 3, seniorconsultant] - I would say that I'm as good or even better than thesystem. I don't feel the need to rely on it; I'll just dowhat I do. We are all trained endocrinologists, so wetrust our judgment because that has been our breadand butter for many years. At the end of the day, webear the responsibility for our patients, so you know,if the algorithm makes a sound decision, butsomething unfortunate ever happens to the patient,then it's still our own accountability on the line. [FGD1, consultant] - Some of the recommendations go against your clinical judgement. For example, I have two patients and theAI recommendation was to add a beta blocker tosomeone who doesn't have ischemic heart disease asa second line agent. That's just not something that we would normally do. So have to exercise caution too![FGD 1, consultant] - These recommendations would be more valuable in a primary healthcare setting, where doctors may not have extensive knowledge of clinical practices related to novel glucose-lowering medications and insulin titration, especially in complex cases. I think implementing the AP tool in such settings would greatly help doctors in improving patient engagement and care. [FGD 1, consultant] |
| Zheng et al.2024[4] | - Improving the transparency and explainability of AI tools is essential for building trust among clinicians. | - Some patients probably have asthma that we don'tdetect, but that's where I think this tool would behelpful because maybe even though they don't havea diagnosis of asthma, they've had wheezing, or otherthings listed in their diagnosis and problem list. Thatwould be helpful to avoid missing those people. [P6] - Some kids had been given a bronchodilator becauseoften at 18 months, they present with like viralinduced wheezes, and we find it improves withalbuterol. So, we get a response to albuterol, and weknow that these kids are potentially likely to getasthma, but we typically don't make that diagnosisuntil after two. [P4] - If we are getting this risk score and especially if itwere telling them that this is somebody that is at highrisk of relapses and recurrences of episodes, then wecan make that effort to reach out to thoseindividuals. That should be flowing in my mind. Thatshould be going to our care teams. [P14] - I want to be able to see that risk score. When the patient is in front of me, I also want to be able to see a whole lot more information about thatpatient, preferably in an easy to find format that I don't have to go digging in Epic for it, like I currently do. [P14] - I would probably like something simpler, like not necessarily a percentage. And then I like, okay, it's red, which means they're at high risk. Inthe background, I could know what that means. And if you want more information, then you could click and find why it is high. [P13] - I think high, medium, low would, you know, would be sufficient. And if you would have something popping up or even color-coded too, like theyare low risk in green, medium in yellow. If they're high-risk and in red, that certainly will get your attention. I also want to know what is puttingthem at risk. Is it the severity of symptoms, their need for oral steroids, their hospitalization and ED visits? So that would certainly be helpful toknow exactly where their risk area is. [P9] - This prediction score is not meant to override. This is complimentary information for you. I know you do mental calculations, but this is adata-driven calculation that gives you other complementary information. If there's a discrepancy, is there anything you are thinking low inemotion, say “hi, just to think about it on this page.” So then, you know, you don't have to go to that page, just look through another page of thesectional summary. [P5] - Parents may worry about their child if the AI tool says, “high risk of AE” and subsequently change daily decisions, such as not sending their child to school or letting them play outside. [P7] |
| Elareed et al.2024[38] | - AI can support healthcare efficiency and workload reduction, but ethical and practical concerns need to be addressed for broader acceptance | - "Nearly 40% of participants disagreed that physicians will not fight for patient's life in case AI predicts low chance of survival for the patient. - Nearly 65% of participants agreed that AI will enhance patient care by making more data available for research and around 58% agreed with the opinion that AI-based decision support systems must be scientifically proven before using. - 44.2% of participants agreed that technical malfunction by AI is more serious than wrong decision by a physician. - More than half of participants agreed that AI will save time for physicians to deal more with patients. Around 40% of patients agreed that physicians should have the final control over diagnosis of patients and agreed that the use of AI impairs physician-patient relationship. - More than half of participants agreed that AI use will reduce the overload of physicians. " |
| Vereschak et al.2024[11] | - Understanding the perspectives of different stakeholders is essential for designing effective Human-AI interactions that foster trust. | - Positive expectations and perceived risk are prerequisites for the emergence of trust, but the nature of risk is debated “It is important that the owner [of an AI-embedded system] does not recommend something in the company’s interest” (P6). - Perceived risk associated with a decision as another prerequisite for the emergence of trust: “When my physical integrity or money is at risk, trust becomes a consideration, especially when something important is at stake for me” (P4).“... a foundation [for defining risk] would be the physical needs and individual and social integrity from the Maslow’s Hierarchy.” However, some, like P5 and P2, broaden the concept of risk to include “vulnerability” (P5) or “responsibility” (P2) - Task complexity as a new prerequisite for the emergence of Human-AI trust.“Sometimes you can’t evaluate everything, you sort of use that quick «I just trust you, I just trust you to do the right thing».” - Trust is differentiated from trust-related behaviors and trustworthiness.“can have a complex and elaborate way of thinking [about AI-embedded systems and recommendations]” (P4)“as long as there aren’t too many complaints, no negative comments, [...] and the user uses the solutions, we can consider that trust is not broken” (P2).“For me, it [trustworthiness] is not so much a question of AI, it’s more between the individual and the entity or the organization that makes the system.” - The team behind AI plays an important role in (Human-AI) trust."[...] is established before the system exists. [...] Trust is very strong in the co-design phase [between users and the AI team]” (P4).“We have 10,000 users, and 90% of them say «the feedback from the AI was very interesting», now [knowing this, current users] will tend to trust the AI” (P6). This trust in AI is further strengthened if “a domain expert confirms what the AI recommends” (P6). - “There is trust in the system and trust in those who use the system [...]. They [the users] should at least tell you they are using such a system [embedding AI] so you don’t lose your chance, just because you don’t know how it works [...]”.“I don’t trust mixing humans and machines. Either the decision should be entirely made by a machine or a human. If you have only one machine, then you know what to expect. But if you have a machine and a human, then it would be very unfair because the users’ roles are not defined, and the priority is not clear." - “[he] is building trust with people through his own presence in the media [...]. People trust him and love his personality, so they trust his product even if it does not benefit them in the end.” - The effect of AI certification on Human-AI trust depends on who is behind it.“AI certificates are very important [for Human-AI trust] if there are organizations [that issue them] that people can trust” (DS2) |
